# Foot Structure in Boys with Down Syndrome

**DOI:** 10.1155/2017/7047468

**Published:** 2017-08-21

**Authors:** Ewa Puszczałowska-Lizis, Krzysztof Nowak, Jarosław Omorczyk, Tadeusz Ambroży, Przemysław Bujas, Leszek Nosiadek

**Affiliations:** ^1^Institute of Physiotherapy, Faculty of Medicine, University of Rzeszow, Warszawska 26A Street, 35-205 Rzeszow, Poland; ^2^Special Purpose School and Education Center, Mrowla 79C, 36-054 Mrowla, Poland; ^3^Institute of Sport, Faculty of Physical Education and Sport, University of Physical Education in Krakow, 78th Jan Pawel II Avenue, 31-571 Krakow, Poland; ^4^Institute of Biomedical Sciences, Faculty of Physical Education and Sport, University of Physical Education in Krakow, 78th Jan Pawel II Avenue, 31-571 Krakow, Poland

## Abstract

**Introduction and Aim:**

Down syndrome (DS) is associated with numerous developmental abnormalities, some of which cause dysfunctions of the posture and the locomotor system. The analysis of selected features of the foot structure in boys with DS versus their peers without developmental disorders is done.

**Materials and Methods:**

The podoscopic examination was performed on 30 boys with DS aged 14-15 years. A control group consisted of 30 age- and gender-matched peers without DS.

**Results:**

The feet of boys with DS are flatter compared to their healthy peers. The hallux valgus angle is not the most important feature differentiating the shape of the foot in the boys with DS and their healthy peers. In terms of the V toe setting, healthy boys had poorer results.

**Conclusions:**

Specialized therapeutic treatment in individuals with DS should involve exercises to increase the muscle strength around the foot joints, enhancing the stabilization in the joints and proprioception. Introducing orthotics and proper footwear is also important. It is also necessary to monitor the state of the foot in order to modify undertaken therapies.

## 1. Introduction

The human foot is an important part of the static-dynamic motor organ and is shaped uniquely in each individual. Its construction and setting have major impacts on the quality of gait and postural stability. A properly arched foot is elastic and flexible and absorbs microtrauma and shocks during locomotion, making the gait light and springy [1]. This state is conditioned by the proper capacity of muscles and ligaments and proper construction of the osteoarticular system.

Down syndrome (DS), also known as trisomy 21, is a genetic disorder caused by the presence of all or part of a third copy of chromosome 21 [2]. It is associated with characteristic symptoms and physical features recognized in the human since the moment of the birth [3]. Developmental abnormalities, muscular hypotonus, and excessive flexibility of the ligamentous articular system cause postural problems in these individuals. Musculoskeletal function, postural stability, and coordination skills may be compromised or impaired [4–8]. Because the quality of the gait is reduced in individuals who have DS, their physical activity is often restricted [9, 10]. Cioni et al. [11] observed that strength of the main antigravity muscles, the knee extensors in children and adolescents with Down syndrome, is markedly affected during the execution of slow isokinetic movements. Bolach et al. [12] emphasized that the degree of intellectual disability has a major impact on the results of motor capacity tests. The examination by means of the Eurofit Special test (while being a battery of motor fitness tests resulting from a 10-year project of the Committee of Experts for Sports Research and being comprised of strength, speed, flexibility, and balance) showed worse results in children aged 11 to 14 years with moderate intellectual disabilities compared to their peers with mild mental disabilities. An additional issue for people with DS is their predisposition to excessive weight gain resulting from genetic condition, metabolic and hormonal disorders, deficiency of movement, and an inability to diagnose their own nutritional needs [13–15]. These aspects often determine the emergence and deterioration of pathological changes in the structure of the feet that are difficult to treat and rehabilitate.

The aim of this study was the analysis of selected features of the foot structure in boys with DS versus their age- and gender-matched peers without Down syndrome.

## 2. Materials and Methods

The study included 30 boys with DS (16 were 14-year-olds and 14 were 15-year-olds) attending the Special Purpose School and Education Center in Mrowla, the Special Purpose School and Education Center in Ropczyce and UNICEF Special Schools Complex in Rzeszow (Poland). The inclusion criteria were a confirmed genetic diagnosis of Down syndrome by a pediatric neurologist, age between 14 and 15 years, physical fitness that allowed for walking without orthopedic equipment, and the ability to take a standing position on the podoscope independently. In addition, understanding the instructions that were necessary for the measurement procedures and written consent of parents or guardians to participate in the study were also criteria. The exclusion criteria included a previous orthopedic surgery. A control group consisting of 30 age- and gender-matched peers attending junior high school in the School Complex in Swilcza (Poland) without Down syndrome or cognitive disorders and without signs of orthopedic disease were also recruited for this study.

**Figure 1:**
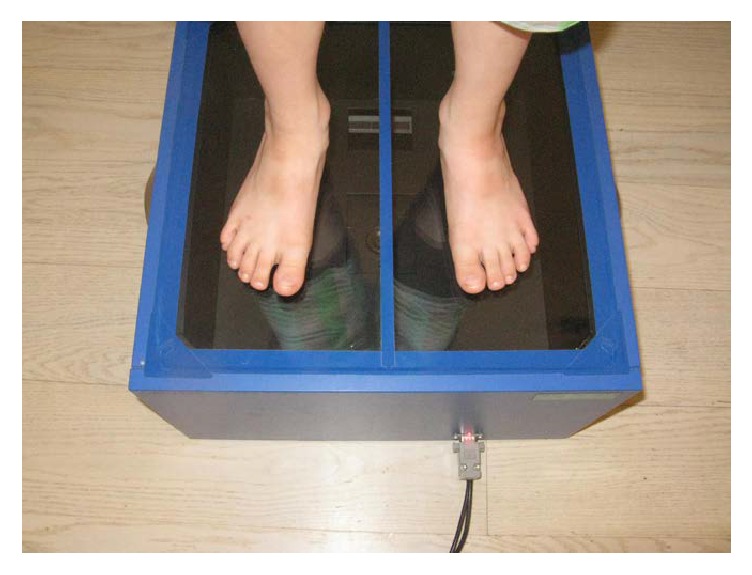
Podoscopic survey sample. Source: own study. The authors obtained the participant's parent consent to publish the image.

The CQ-ST podoscope (manufactured by Electronic System) was used as the main research tool. The podoscopic examination of the plantar side of the foot is the development and improvement of the well-known plantographic method. In addition to an exact foot print, we obtained information about the foot arching. The study entailed measuring of the plantar feet surfaces in a relaxed stance, with the upper limbs hanging along the body. Each time, both feet were subjected to examination. The width and foot angle were natural, unforced (Figure 1). Based on the picture obtained during the scan, the researcher marked by hand specific points in computer and next, on the basis of those points, the computer calculated the indices describing the longitudinal and transverse arch of the foot and arrangement of the hallux and the fifth toe. All footprints were elaborated by the same person.

The following parameters were measured:

- foot length: the length of the segment connecting the most distal point of the forefoot (on the pad of the longest toe) with the farthest point within the hindfoot, in cm;
- foot width: the length of the segment connecting the most medially located point on the head of the first metatarsal bone (metatarsale tibiale: mtt) with the point lying most laterally on the head of the V metatarsal bone (metatarsale fibulare: mtf) in cm;
- Clarke's angle: constructed by drawing a tangent to the medial edge of the foot (the prints) and a line connecting the deeper part of the footprint with the most medial point of the forefoot, in degrees;
- the Wejsflog (*W*) index: the ratio of the length to the width of the foot;
- hallux valgus angle (*α*): the angle between the tangent to the medial edge of the foot and a tangent drawn from the point at the widest part of the forefoot (mtt) to the outer edge of the hallux, in degrees;
- the angle of the varus deformity of the fifth toe (*β*): the angle between the tangent to the lateral edge of the foot and a tangent drawn from the point at the widest part of the forefoot (mtf) to the outer edge of the V finger, in degrees.

**Figure 2:**
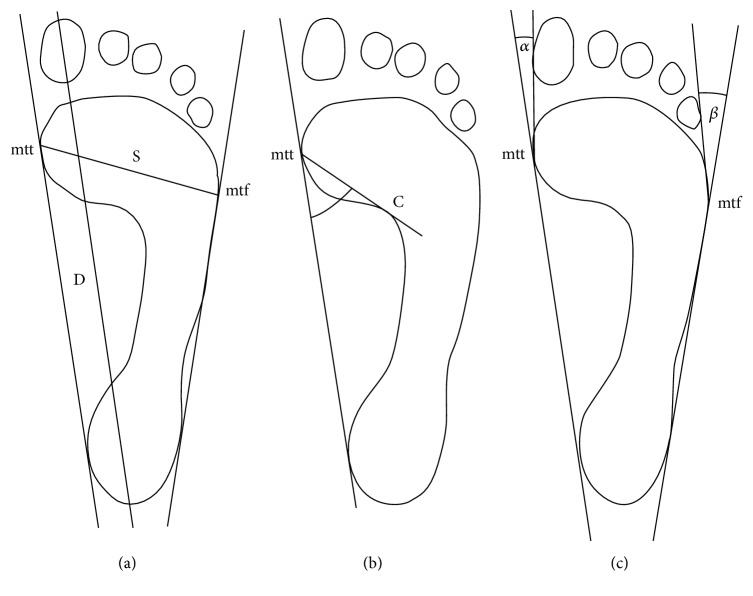
Procedure for determining the feet structure indices: (a) foot length (*D*), foot width (*S*), and the Wejsflog (*W*) index; (b) Clarke's angle; (c) hallux valgus angle (*α*) and the angle of the varus deformity of the fifth toe (*β*).

The procedures for calculating the feet structure indices are shown in Figure 2.

Anthropometric measurements of the body mass and height were taken. The body mass was measured with electronic scales, determined to the nearest 0.1 kg. The body height was measured to the nearest 0.1 cm using a Martin-type anthropometer. The obtained data were used to calculate BMI. Basic descriptive statistics of the somatic features in the examined boys are presented in Table 1.

In order to preserve the integrity of the research process, all the measurements were taken in the gym, in the morning, using the same measuring instruments operated by the authors. Boys were wearing their gymnastic uniforms without shoes. Procedures were carried out in accordance with Declaration of Helsinki for experiments involving humans. All participants, their parents, or legal guardians received detailed information concerning the aim and methodology used in the study. The study was approved by the Ethical Review Board of the Rzeszow University (number 6/01/2015) and performed after obtaining written consent from the children's parents or legal guardians.

Based on the accumulated data, the following descriptive statistics were calculated: arithmetical mean value $\left(\overset{-}{x}\right)$, standard deviation (SD), median (Me), and maximum and minimum values were given. The consistency of the values with the normal distribution was verified by means of the Shapiro-Wilk test. In order to evaluate intergroup differences in average level of tested variables, we used the Student's *t*-test for independent samples or, alternatively, the nonparametric Mann–Whitney *U* test. The results were considered statistically significant, if the probability level of the test was lower than the predetermined significance level *p* < 0.05. The Stat Soft STATISTICA application (version 10.0) was used to process the test results.

## 3. Results

Table 2 shows the descriptive statistics of selected parameters of the feet structure in the examined boys. These data show that the boys with DS were characterized by significantly shorter and narrower feet compared to their peers without developmental disorders. Considering the level of the longitudinal arch, it should be noted that the average value of Clarke's angle in boys with DS was lower in both the right and the left foot. The analysis of Clarke's angle indicates reduction in the longitudinal arch of the foot while comparing to the norms, which, according to Lizis [1], are 32° to 47° for the 14-year-olds and 36° to 50° for the 15-year-olds. The intergroup comparison of the value of Wejsflog's index (*W*) showed a statistically significant lower value in the boys with DS. The average hallux valgus angle (*α*) did not deviate from the norms and was similar for the right and left foot. No statistically significant intergroup differences in the values of this index were found, which indicates that these are not the most important characteristics differentiating the construction of the feet in boys with DS and their healthy peers. The average values of the varus angle of the V toe (*β*) in boys with DS were within the normal range, which was adopted at the variation range from 0° to 9°, whereas in the control group they were above the upper limit of normal [16]. The intergroup comparisons showed statistically significant differences in the values of this parameter.

## 4. Discussion

Issues concerning the construction of the foot have been repeatedly discussed by various authors. Many of them highlighted the correlation between insufficient movement, improper footwear, and excessive static loads with the foot structure. There are studies in the literature in which authors undertook the issues of foot structure in children and adolescents with developmental disorders. Concolino et al. [4] evaluated the construction of the lower limbs in 50 children with DS, including 19 girls and 31 boys aged 3 to 8 years versus 100 children without DS, including 32 girls and 68 boys matched in age. Physical examination of the lower extremities showed a statistically significant higher incidence of isolated metatarsus primus varus and both hallux valgus and metatarsus primus varus in children with DS. Podoscopic examination has shown far more flat feet and isolated calcaneal valgus in children with DS compared to their healthy peers. The authors emphasize that observed deformations can cause dysfunction of the foot. Mirska et al. [17] underlined the negative impact of joint hypermobility on different parts of the motor system. According to the authors, flat feet is one of the most common symptoms of excessive joint laxity, and most changes occur within the most movable joints: the talocalcaneal and calcaneonavicular joints. Lim et al. [18] analyzed the correlation of foot structure and footwear fitting with disability (determined using the parent-reported Oxford Ankle Foot Questionnaire for Children) in 50 Australian children and adolescents. The group consisted of 22 females and 28 males, aged 5 to 18 years, with DS. The average arch index for the study group was 0.29 ± 0.08. The authors noted 38 flat feet, 5 hallux valgus, and 6 cases of minor toe deformities. It was found that flatter feet and lesser toe deformities are not associated with foot-specific disability in children and adolescents with DS. Hallux valgus is associated with foot-specific disability during school and play activities. Ill-fitting footwear (too narrow) is common and is associated with foot-specific disability. Based on the analysis of the percentage differences between the dimensions of the foot and shoe size worn, the authors observed a mismatch of footwear, which means that the subjects wore too narrow shoes.

The issue of the impact of the degree of intellectual disability on the longitudinal arch of the foot in 80 residents of Special Leeds Education Center in Tarnow (Poland) was described by Jankowicz-Szymanska et al. [15]. The comparison of the results of the research in 40 individuals with mild and 40 individuals with moderate disability (each group consisted of 27 males and 13 females) and the analysis of Spearman's rank correlation coefficients between the degree of intellectual disability and the values of Clarke's angle allowed the conclusion that there is no direct correlation between the degree of intellectual disability and the longitudinal foot arch in young people aged 16 to 22 years. It seems that the difficulty in determining the unequivocal relationship between the mentioned features can result from the coexistence of many disorders characteristic of DS, which overlap making it difficult or even impossible to draw concrete conclusions.

The research for our study showed that the feet of boys with DS are shorter and narrower compared to their healthy peers. There was a clear flattening of the dynamic foot arch. Also, in terms of the transverse arch, determined based on the ratio between the length and width of the foot, the boys with DS achieved worse results. The cause of the reduction of longitudinal and transverse arch rates may be abnormal function of the muscles and ligaments and excessive body weight. In turn, the comparison of the hallux valgus angle showed no intergroup differences and, more important, the average values of these angles are within the normal range in both groups. This leads to the conclusion that hallux valgus angle is not the most important feature differentiating the shape of the foot in the boys with DS and their healthy peers. It is worth noting that, in terms of the V toe setting, boys in the control group had poorer results. The tendency to a more pronounced varus of the V toe may be due to the increased load on the edge side of the foot. The analysis of literature related to the subject allowed us to note that most authors focused on assessing the formation of the longitudinal foot arch and hallux settings. This article is an attempt to conduct a comprehensive analysis of foot shape in boys with DS. Efforts were made to make the groups uniform, particularly for age (the results were analyzed separately for 14- and 15-year-olds) and gender. This approach justifies Demczuk-Włodarczyk's [16] observations, namely, that dimorphism of longitudinal architecture in feet is clear from an early age and affects the arch height, the pace of their development, and the symmetry of shape.

Analysis of an extensive literature and the results of feet research point to the need to refer children and adolescents with DS to appropriate therapeutic procedures, which should include exercises aimed at strengthening the muscle around the foot joints and improving the stability of joints and proprioception, starting with static tasks and then adding exercises in dynamic conditions. The foot is one of the links in the proprioceptive kinetic chain of a man. Therefore, activities should be focused on exercises affecting the entire organ of movement and body posture. Improvement should take into account such specialized methods as Proprioceptive Neuromuscular Facilitation (PNF), kinetic control, and sensory integration, as well as general fitness exercises, swimming, and physical therapy. It is important to implement orthotics and proper footwear to stabilize and relieve overburdened parts of the feet. It is necessary to constantly monitor the state of the foot in order to modify the therapeutic treatment as needed. According to a holistic approach to the patent and his complex problems, physiotherapeutic issues should be treated in an interdisciplinary way. This implies the need to connect a variety of methods of enhancing motor skills, psychopedagogy, and various forms of social adaptation. The influence of environmental factors is equally important, especially through the education of families regarding proper diet of the patient, the importance of daily physical activity, and the selection of appropriate footwear.

## 5. Conclusions

The feet of boys with DS are shorter and narrower and longitudinally and transversely flatter compared to the feet of healthy peers. The hallux valgus angle is not the most important feature differentiating the shape of the foot in boys with DS and their healthy peers. In terms of the V toe setting, boys in the control group showed poorer results. Specialized therapeutic treatment in children and adolescents with DS should primarily involve exercises to increase the muscle strength around the foot joints, enhancing the stabilization in the joints and proprioception, as well as introducing orthotics and proper footwear. It is also necessary to constantly monitor the state of the foot in order to modify undertaken therapeutic conduct.

## Figures and Tables

**Table 1:** Comparison of somatic features recorded for the DS group and control group.

Age [years]	DS group			Control group			*t*/*U*	*p*
	$\overset{-}{x}\pm SD$	Me	Max–min	$\overset{-}{x}\pm SD$	Me	Max–min		
Body weight [kg]								
14	49.56 ± 10.11	46.50	67.00–33.00	56.06 ± 12.85	53.00	81.00–35.00	*t* = −1.59	0.122
15	68.43 ± 6.91	69.50	81.00–58.50	63.39 ± 9.17	58.25	85.00–55.00	*U* = 55.50	0.041^*∗*^

Body height [cm]								
14	149,44 ± 6,81	151.50	157.00–137.00	170.06 ± 9.84	171.00	185.00–149.00	*U* = 16.00	<0.001^*∗*^
15	164,57 ± 5,46	164.00	172.00–155.00	176.86 ± 4.93	176.50	184.00–164.00	*U* = 7.50	<0.001^*∗*^

BMI								
14	22.24 ± 4.48	20.91	30.47–17.31	19.25 ± 3.53	17.84	27.01–15.58	*U* = 70.00	0.029^**∗**^
15	25.28 ± 2.52	25.97	28.49–20.03	20.26 ± 2.73	18.50	26.81–18.15	*U* = 21.00	0.002^*∗*^

^*∗*^ *p* < 0.05.

**Table 2:** Comparison of foot structure parameters recorded for the DS group and control group.

Age [years]	DS group			Control group			*t*/*U*	*p*
	$\overset{-}{x}\pm SD$	Me	Max–min	$\overset{-}{x}\pm SD$	Me	Max–min		
Length of the right foot [cm]								
14	20.98 ± 0.98	20.90	22.50–19.30	24.40 ± 1.34	24.35	26.50–21.50	*t* = −8.26	<0.001^**∗**^
15	23.12 ± 1.59	23.25	26.00–20.50	25.39 ± 0.60	25.20	26.90–24.70	*U* = 19.00	<0.001^*∗*^

Length of the left foot [cm]								
14	20.86 ± 0.92	20.60	22.50–19.40	24.48 ± 1.35	24.20	26.90–21.50	*t* = −8.88	<0.001^*∗*^
15	23.14 ± 1.69	23.10	26.50–20.90	25.52 ± 0.74	25.30	26.90–24.50	*t* = −4.82	0.001^*∗*^

Width of the right foot [cm]								
14	8.27 ± 0.64	8.45	9.20–7.10	8.89 ± 0.89	8.90	10.70–7.50	*t* = −2.26	0.031^**∗**^
15	8.62 ± 0.50	8.70	9.30–7.50	8.87 ± 0.67	8.90	9.90–8.00	*t* = −1.12	0.274

Width of the left foot [cm]								
14	8.42 ± 0.55	8.60	9.30–7.20	8.99 ± 0.78	8.90	10.30–7.50	*t* = −2.38	0.024^**∗**^
15	9.01 ± 0.70	9.10	10.10–7.60	9.03 ± 0.70	9.05	10.20–8.00	*t* = −0.05	0.958

Clarke's angle of the right foot [°]—the medial longitudinal foot arch								
14	21.06 ± 11.32	20.50	38.00–4.00	42.88 ± 9.46	41.00	56.00–16.00	*U* = 11.00	<0.001^*∗*^
15	20.36 ± 9.99	18.50	34.00–3.00	38.79 ± 8.32	40.00	50.00–20.00	*t* = −5.30	<0.001^*∗*^

Clarke's angle of the left foot [°]—the medial longitudinal foot arch								
14	19.50 ± 9.65	19.50	38.00–3.00	41.81 ± 9.81	40.50	55.00–14.00	*U* = 13.50	<0.001^*∗*^
15	19.00 ± 12.30	19.00	50.00–4.00	36.50 ± 6.45	37.00	45.00–18.00	*U* = 20.50	<0.001^*∗*^

Wejsflog (*W*) index of the right foot—the transverse foot arch								
14	2.54 ± 0.22	2.55	2.87–2.10	2.75 ± 0.22	2.71	3.00–2.30	*t* = −2.75	0.010^**∗**^
15	2.68 ± 0.17	2.73	2.92–2.36	2.84 ± 0.16	2.85	3.00–2.60	*U* = 48.50	0.021^**∗**^

Wejsflog (*W*) index of the left foot—the transverse foot arch								
14	2.48 ± 0.19	2.47	2.86–2.20	2.73 ± 0.18	2.79	3.00–2.35	*t* = −3.72	0.001^**∗**^
15	2.57 ± 0.16	2.59	2.79–2.27	2.81 ± 0.16	2.84	3.00–2.59	*U* = 32.50	0.002^**∗**^

Hallux valgus angle (*α*) of the right foot [°]								
14	5.81 ± 5.82	4.00	20.00–0.00	3.12 ± 4.47	1.50	16.00–0.00	*U* = 83.50	0.094
15	3.36 ± 3.63	2.00	10.00–0.00	3.79 ± 3.26	4.00	9.00–0.00	*U* = 91.00	0.769

Hallux valgus angle (*α*) of the left foot [°]								
14	7.81 ± 6.36	6.50	25.00–0.00	4.00 ± 4.56	2.00	14.00–0.00	*U* = 77.50	0.056
15	6.07 ± 6.44	4.00	22.00–0.00	5.64 ± 4.18	5.50	13.00–0.00	*U* = 92.50	0.804

The V toe varus deformity angle (*β*) of the right foot [°]								
14	4.00 ± 5.72	0.00	16.00–0.00	15.25 ± 7.06	17.00	24.00–0.00	*U* = 31.50	<0.001^*∗*^
15	9.93 ± 5.08	9.50	18.00–0.00	15.64 ± 6.82	16.50	26.00–0.00	*t* = −2.51	0.018^**∗**^

The V toe varus deformity angle (*β*) of the left foot [°]								
14	3.13 ± 4.99	0.50	17.00–0.00	13.25 ± 6.41	13.00	23.00–0.00	*U* = 29.50	<0.001^*∗*^
15	7.00 ± 7.37	7.50	22.00–0.00	14.79 ± 5.04	14.00	26.00–6.00	*U* = 37.00	0.004^**∗**^

^*∗*^ *p* < 0.05.
